# Genetic Variations in Vitamin D Metabolism Linking Epigenetic Regulation and Microbiota Interactions: From Phenotype to Genotype

**DOI:** 10.7759/cureus.98298

**Published:** 2025-12-02

**Authors:** Gulsen Meral, Muhammed Yunus Alp, Neval Burkay, Esma Gökcen Alper Acar, Bilge Ozluer Baser, Selen Baran Ozmen, Ruya Atesli, Ece Aydin, Elif S Aslan

**Affiliations:** 1 Medical Genetics, Epigenetic Coaching, Norwich, GBR; 2 Pediatrics, Faculty of Medicine, Istanbul Nisantasi University, Istanbul, TUR; 3 Medical Genetics, Genoks Genetics Center, Ankara, TUR; 4 Medical Genetics, Epigenetic Coaching, Istanbul, TUR; 5 Nutrition, Epigenetic Coaching, Mugla, TUR; 6 Medical Biology, Epigenetic Coaching, London, GBR; 7 Statistics, Mimar Sinan Fine Arts University, Istanbul, TUR; 8 Pediatrics, Acibadem Kent Hospital, Izmir, TUR; 9 Molecular Biology and Genetics, Faculty of Engineering and Natural Sciences, Biruni University, Istanbul, TUR

**Keywords:** epi̇geneti̇c, polymorphism, public health, vdr, vitamin d

## Abstract

Background: Vitamin D deficiency is a global health concern affecting all age groups, despite the implementation of supplementation strategies. This study aimed to investigate the roles of genetic polymorphisms, epigenetic mechanisms, and microbiota interactions in vitamin D metabolism, as well as their potential contributions to allergies, autoimmune thyroiditis, and autism spectrum disorder.

Methods: This retrospective, descriptive study analyzed data from 60 individuals (16 males and 44 females) who underwent nutrigenetic testing between 2022 and 2024 at Epigenetic Coaching, Istanbul, Turkey. Genetic analyses focused on the cytochrome P450 family 2 subfamily R member 1 (CYP2R1) gene, group-specific component (GC) gene, and vitamin D receptor (VDR) polymorphisms. Inclusion criteria included the presence of allergic symptoms, a diagnosis of autism spectrum disorder, or a diagnosis of Hashimoto’s thyroiditis, along with prior genetic testing for the selected variants. Individuals with any known genetic disorder other than the studied variants were excluded.

Results: CYP2R1, GC, and VDR polymorphisms associated with vitamin D insufficiency were found at higher frequencies in patients with autism, Hashimoto’s thyroiditis, and allergic conditions. When examining the associations between genetic variants and diseases, VDR FokI, VDR ApaI, and VDR BsmI variants were more frequently observed in patients with Hashimoto’s thyroiditis, while the VDR TaqI variant was more common in individuals with autism. In the allergy group, BsmI, FokI, ApaI, and TaqI variants were identified at higher frequencies.

Conclusion: Genetic variants and epigenetic modifications may disrupt vitamin D metabolism and alter gut microbiota diversity, contributing to the development of various diseases. Evaluating vitamin D deficiency together with its genetic, epigenetic, and microbiota determinants is essential for developing effective prevention and treatment strategies. These findings emphasize the necessity of personalized, nutrigenetic-based approaches to optimize vitamin D function and support overall health.

## Introduction

Vitamin D deficiency is a significant global health concern that affects individuals across all age groups. It remains prevalent even in regions with adequate ultraviolet radiation and in countries where supplementation programs are implemented [1,2]. The lack of comprehensive data for specific populations, particularly infants, children, adolescents, and pregnant women, poses challenges to the effective management of this condition [1].

Although vitamin D has been traditionally associated with bone health, growing evidence indicates that it also plays critical roles in cell differentiation, vascular function, and metabolic regulation [3]. Moreover, vitamin D exerts important immunomodulatory effects. Its interaction with T lymphocytes and the demonstration of local synthesis within immune cells highlight its relevance in autoimmune, inflammatory, and infectious diseases. Supplementation has shown potential benefits in specific conditions such as rheumatoid arthritis and inflammatory bowel disease; however, individual responses may vary due to underlying genetic differences. Therefore, further research is needed to personalize and optimize vitamin D treatment protocols [4-8].

The metabolism of vitamin D is regulated through the interplay of genetic and epigenetic factors. Three key genes play pivotal roles in this process: the cytochrome P450 family 2 subfamily R member 1 (CYP2R1) gene encodes the enzyme responsible for hepatic 25-hydroxylation of vitamin D; the group-specific component (GC) gene encodes the vitamin D-binding protein (DBP); and the vitamin D receptor (VDR) gene mediates the biological effects of vitamin D through receptor activation. Polymorphisms in these genes can contribute to interindividual variability in vitamin D status [4,7].

Genetic and epigenetic factors together shape the biological actions of vitamin D, influencing both an individual’s response to supplementation and the severity of health risks associated with deficiency. Environmental determinants, particularly nutrition and sunlight exposure, also play a crucial role in regulating vitamin D-mediated gene expression [9]. Understanding this complex network of biological interactions is essential to elucidating how genetic variations in vitamin D-related pathways contribute to different phenotypic outcomes.

Vitamin D also supports intestinal integrity and the maintenance of host-microbiota balance. Through the VDR pathway, it promotes epithelial cell survival, strengthens the mucosal barrier, and enhances the production of antimicrobial peptides. These effects help preserve intestinal homeostasis by limiting bacterial translocation. Disruption of this balance (dysbiosis) has been associated with autoimmune, inflammatory, and metabolic diseases [4].

Our study aims to assess the impact of polymorphisms in vitamin D-related genes on the prevalence of diseases such as Hashimoto’s thyroiditis, allergic disorders, and autism spectrum disorder (ASD). In conjunction with the existing literature, it seeks to elucidate the complex interplay between vitamin D, genetics, epigenetics, and the microbiome. This multidimensional approach may provide novel insights into addressing vitamin D deficiency as a persistent global public health challenge.

## Materials and methods

Study design and ethical approval

This retrospective and descriptive study was conducted in accordance with the principles of the Declaration of Helsinki. The study protocol was reviewed and approved by the Ethics Committee of Biruni University, Istanbul, Turkey (approval no: 2024-BIAEK/06-41).

Prior to genetic testing, written informed consent was obtained from all participants or their legal guardians in accordance with institutional and ethical principles.

Study population

Participants were identified through a retrospective data analysis of individuals who underwent genetic testing at Epigenetic Coaching, Istanbul, Turkey, between 2022 and 2024. A total of 60 individuals (16 males and 44 females), aged between six and 70 years, were included in the study.

The inclusion criteria were defined as follows: presence of allergic symptoms, diagnosis of ASD or Hashimoto’s thyroiditis, and availability of genetic test results for variants in the CYP2R1, GC, and VDR genes. Exclusion criteria included the presence of additional diagnosed genetic, metabolic, or chronic diseases beyond the three conditions under study (allergic disease, ASD, or Hashimoto’s thyroiditis), as well as a lack of genetic test data for the relevant variants.

Genetic analysis

Genomic DNA was extracted from oral epithelial cell swabs using the phenol-chloroform extraction method. DNA concentration and purity were assessed using a NanoDrop™ 1000 spectrophotometer (Thermo Fisher Scientific, Wilmington, USA) and standardized to 50 μg/mL.

Samples were analyzed using the Illumina iScan platform (Illumina, San Diego, CA, USA) with a custom-designed Infinium HTS iSelect microarray. Single-nucleotide polymorphisms (SNPs) were identified using Illumina GenomeStudio v2.0.5 software (Illumina, Inc., San Diego, CA, USA) [5].

Quality control was ensured by assessing DNA purity (A260/A280 ratio within the range of 1.8-2.0) and by including repeated samples and control genotypes in each analysis batch. The concordance rate between repeated samples was above 99%.

Variables and outcome measures

The main variables assessed were CYP2R1, GC, and VDR gene variants, which play key roles in vitamin D activation, transport, and receptor function. Rather than focusing on serum vitamin D levels, this study aimed to evaluate the potential associations between these genetic variants and clinical phenotypes representative of immune, neurodevelopmental, and autoimmune processes, specifically, allergic manifestations, Hashimoto’s thyroiditis, and ASD.

Statistical analysis

All statistical analyses were performed using IBM SPSS Statistics software, version 27.0 (IBM Corp., Armonk, NY, USA). Descriptive statistics were presented as frequencies and percentages. Genotype frequency distributions among the allergic disease, Hashimoto’s thyroiditis, and ASD groups were compared using the chi-square (χ²) test. In cases where expected cell counts were low, the Monte Carlo exact method was applied to improve the reliability of the results. A p-value < 0.05 was considered statistically significant.

Given the limited sample size and exploratory nature of the analysis, no corrections for multiple comparisons (e.g., Bonferroni adjustment) were applied. Accordingly, p-values are presented unadjusted and interpreted within an exploratory context.

## Results

A total of 60 individuals (16 males and 44 females) were included in the study. Among the participants, 38 had allergic symptoms, 12 were diagnosed with ASD, and 10 had a diagnosis of Hashimoto’s thyroiditis. The baseline demographic characteristics of the diagnostic groups are summarized in Table 1.

**Table 1:** Baseline demographics of the study population N: number of patients; SD: standard deviation

Disease Group	N	Age (years, Mean)	Age (years, SD)	Male	Male (%)	Female	Female (%)
Allergy	38	38.97	15.73	8	21.1	30	78.9
Autism spectrum disorder	12	8.92	1.83	8	66.7	4	33.3
Hashimoto's thyroiditis	10	49.4	6.48	0	0	10	100
Total	60	34.7	18.6	16	26.7	44	73.3

To further explore potential genetic patterns among these groups, genotype distributions of vitamin D-related polymorphisms were analyzed (Table 2). Participants’ genotypes were evaluated based on polymorphisms in genes encoding key enzymes involved in vitamin D metabolism.

**Table 2:** Genotype distributions of CYP2R1, GC, and VDR polymorphisms in patients with allergy symptoms, autism spectrum disorder, and Hashimoto’s thyroiditis. Values are presented as n (%) VDR: vitamin D receptor; CYP2R1: cytochrome P450 family 2 subfamily R member 1; GC: group-specific component (vitamin D–D-binding protein). Risk alleles: T for rs1562902 and rs7041; G for rs2060793; C for rs2282679; C for ApaI (rs7975232); G for BsmI (rs1544410); G for FokI (rs2228570); and C for TaqI (rs731236).

Diagnosis	CYP2R1 rs1562902 (T)	CYP2R1 rs2060793 (G)	GC rs2282679 (C)	GC rs7041 (T)	VDR APA1 rs7975232 (C)	VDR BSM1 rs1544410 (G)	VDR FOK1 rs2228570 (G)	VDR TAQ1 rs731236 (C)
Allergy	CT: 18 (47.4%), TT: 14 (36.8%), CC: 6 (15.8%)	GG: 20 (52.6%), AG: 13 (34.2%), AA: 5 (13.2%)	AA: 24 (63.2%), AC: 13 (34.2%), CC: 1 (2.6%)	GT: 18 (47.4%), GG: 15 (39.5%), TT: 5 (13.2%)	AC: 22 (57.9%), AA: 12 (31.6%), CC: 4 (10.5%)	AG: 23 (60.5%), GG: 10 (26.3%), AA: 5 (13.2%)	GG: 23 (60.5%), AG: 12 (31.6%), AA: 3 (7.9%)	CT: 24 (63.2%), TT: 11 (28.9%), CC: 3 (7.9%)
Autism spectrum disorder	TT: 7 (58.3%), CC: 3 (25.0%), CT: 2 (16.7%)	GG: 7 (58.3%), AA: 2 (16.7%), AG: 3 (25.0%)	AA: 7 (58.3%), AC: 4 (33.3%), CC: 1 (8.3%)	GT: 3 (25.0%), TT: 6 (50.0%), GG: 3 (25.0%)	AC: 7 (58.3%), CC: 2 (16.7%), AA: 3 (25.0%)	GG: 4 (33.3%), AG: 6 (50.0%), AA: 2 (16.7%)	GG: 8 (66.7%), AG: 3 (25.0%), AA: 1 (8.3%)	TT: 4 (33.3%), CT: 6 (50.0%), CC: 2 (16.7%)
Hashimoto's thyroiditis	CT: 6 (60.0%), TT: 3 (30.0%), CC: 1 (10.0%)	AG: 6 (60.0%), GG: 3 (30.0%), AA: 1 (10.0%)	AA: 7 (70.0%), AC: 3 (30.0%)	GG: 5 (50.0%), GT: 3 (30.0%), TT: 2 (20.0%)	AA: 5 (50.0%), CC: 3 (30.0%), AC: 2 (20.0%)	GG: 4 (40.0%), AG: 4 (40.0%), AA: 2 (20.0%)	GG: 6 (60.0%), AG: 3 (30.0%), AA: 1 (10.0%)	TT: 5 (50.0%), CT: 3 (30.0%), CC: 2 (20.0%)

For the CYP2R1 rs1562902 variant, the risk allele T was most frequently observed in the Hashimoto’s group (90%). Similarly, the risk allele G of CYP2R1 rs2060793 showed the highest carrier rate in the Hashimoto’s group (90%). Although the risk allele C of GC rs2282679 was generally less frequent, it was most prevalent in the ASD group (41.6%). The risk allele T of GC rs7041 was also most prominently observed in the ASD group (75%). Overall, analysis of VDR polymorphisms revealed higher frequencies of risk allele carriers in the allergy group (for FokI, BsmI, and TaqI variants), whereas only the ApaI variant showed a higher carrier rate in the ASD group (Table 2).

**Figure 1:**
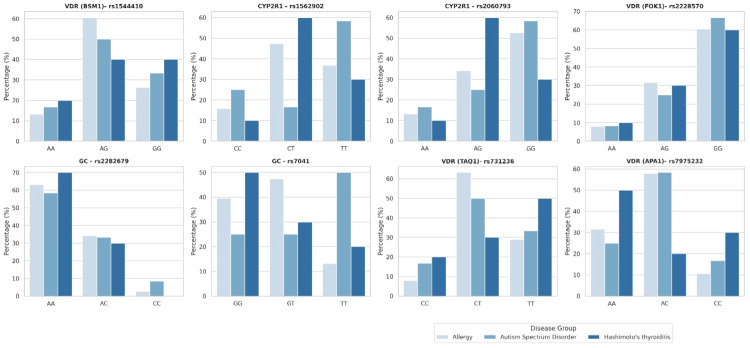
Percentage distributions of genotypes for vitamin D–related polymorphisms (VDR, CYP2R1, and GC) in the allergy, Hashimoto’s thyroiditis, and autism spectrum disorder groups. VDR: vitamin D receptor; CYP2R1: cytochrome P450 family 2 subfamily R member 1; GC: group-specific component (vitamin D–D-binding protein)

To provide a clearer visualization of these findings, Figure 1 presents the percentage distributions of genotypes for each polymorphism across the allergy, Hashimoto’s thyroiditis, and ASD groups.

For CYP2R1 rs1562902, genotype distributions showed moderate variation among groups: the CT heterozygous form was relatively common in both the allergy and Hashimoto’s thyroiditis groups. The TT homozygous genotype was more prevalent in the ASD group, whereas the CC homozygous form was generally rare. A similar trend was observed for CYP2R1 rs2060793; the GG genotype appeared slightly more frequently in the ASD and allergy groups, while the AG heterozygous form predominated in the Hashimoto’s thyroiditis group. Regarding GC polymorphisms, rs2282679 was characterized by the dominance of the AA genotype across all groups, reflecting an overall conserved allele profile. In contrast, rs7041 exhibited subtler variations: the TT genotype was slightly more frequent in the ASD group, the GT heterozygous form was more prominent in the allergy group, and the GG genotype was found at a higher frequency in the Hashimoto’s thyroiditis group. For VDR variants, genotypes of ApaI (rs7975232), BsmI (rs1544410), and TaqI (rs731236) were relatively more frequent in the allergy and ASD groups, suggesting a tendency toward heterozygosity for risk alleles in these populations. In contrast, VDR FokI (rs2228570) exhibited a more balanced and homozygous-dominant distribution across the allergy, ASD, and Hashimoto’s groups, indicating broader genetic diversity rather than group-specific clustering (Figure 1).

Overall, the three diagnostic groups displayed similar genotypic structures. However, subtle yet notable differences were observed, particularly in CYP2R1 and GC polymorphisms within the ASD group and in VDR variants within the Hashimoto’s thyroiditis group. In the allergy group, heterozygous genotypes (such as AG or CT) were more prevalent across most VDR variants (except FokI), suggesting comparatively greater genetic diversity relative to the more homozygous-dominant profiles observed in the Hashimoto’s thyroiditis and ASD groups (Figure 1).

**Figure 2:**
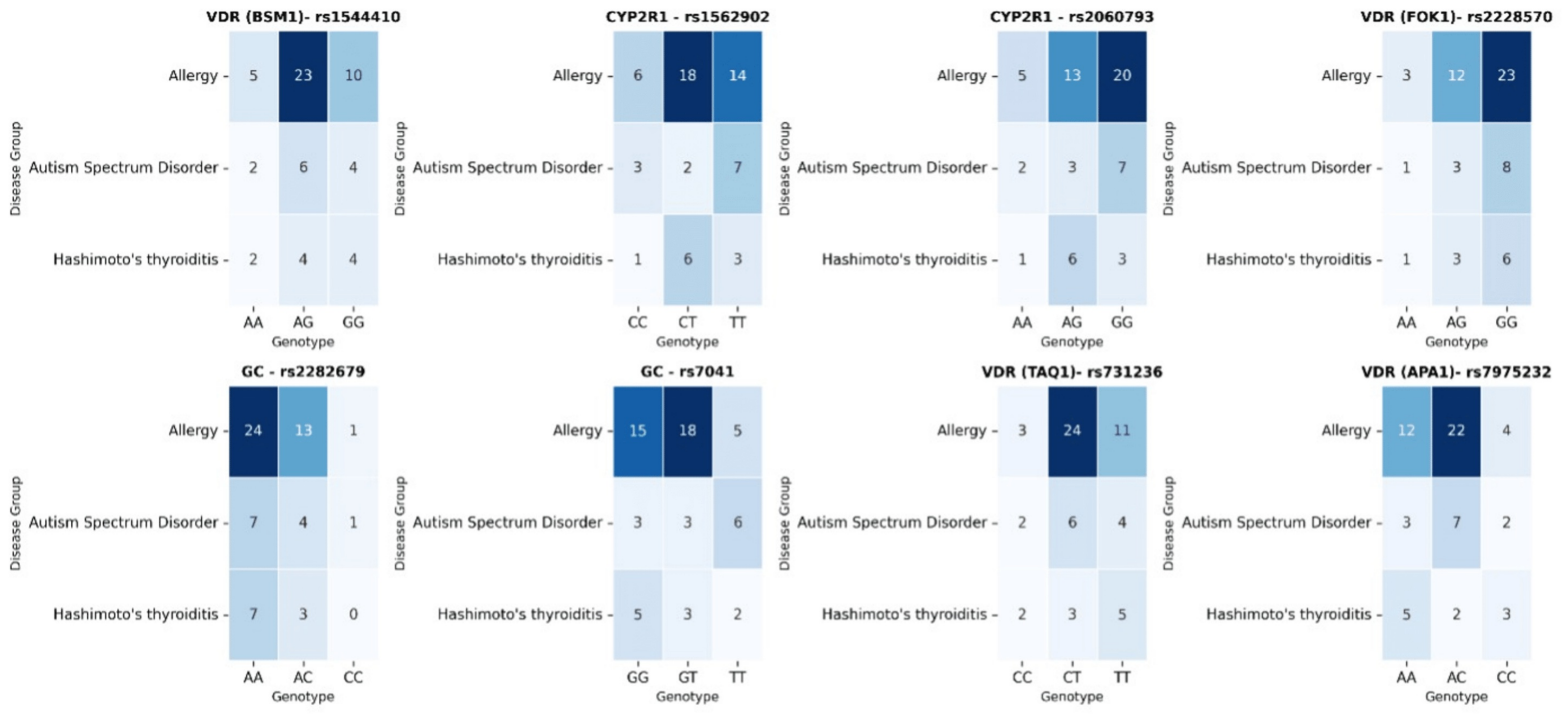
Heatmap visualization of genotype patterns across diagnostic groups VDR: vitamin D receptor; CYP2R1: cytochrome P450 family 2 subfamily R member 1; GC: group-specific component (vitamin D–D-binding protein)

To better assess whether these visual differences reflect underlying genotype clustering, a heatmap visualization was generated (Figure 2).

The heatmap provided a complementary visualization to the bar chart findings. The bar charts revealed mild, group-specific variations, particularly in CYP2R1 and GC polymorphisms within the ASD group and in VDR variants within the Hashimoto’s thyroiditis group. The heatmap, however, confirmed that these patterns were not strong enough to form distinct genotype-based clusters. Both homozygous and heterozygous genotypes were distributed at comparable frequencies across most loci, supporting an overall balanced allele representation. Minor local variations were observed for VDR (BsmI), VDR (FokI), and GC (rs7041); specifically, certain genotype combinations appeared slightly more frequent in the allergy group. Nonetheless, these differences did not reach the level of a clear genotype-based separation among diagnostic groups (Figure 2).

To assess whether the visually observed genotypic differences between diagnostic groups were statistically significant, genotype frequency distributions among the allergy, Hashimoto’s thyroiditis, and ASD groups were analyzed using the χ² test. In cases where expected cell counts were low, the Monte Carlo exact method was applied to enhance reliability (Table 3).

**Table 3:** Chi-square (χ²) analysis of genotype distributions among diagnostic groups (Monte Carlo exact p-values) df: degrees of freedom; p (Monte Carlo): Monte Carlo–derived p value; NS: non-significant; Borderline: borderline significance; VDR: vitamin D receptor; CYP2R1: cytochrome P450 family 2 subfamily R member 1; GC: group-specific component (vitamin D–D-binding protein)

Gene	χ²	df	p (Monte Carlo)	Interpretation
VDR (BSM1) rs1544410	1.499	4	0.847	NS
CYP2R1 rs1562902	4.890	4	0.304	NS
CYP2R1 rs2060793	3.196	4	0.563	NS
VDR (FOK1) rs2228570	0.235	4	0.985	NS
GC rs2282679	1.449	4	0.945	NS
GC rs7041	8.094	4	0.084	Borderline
VDR (TAQ1) rs731236	4.019	4	0.420	NS
VDR (APA1) rs7975232	5.477	4	0.229	NS

The analysis results indicated that none of the examined vitamin D-related polymorphisms showed statistically significant differences among the diagnostic groups (all p > 0.05). However, although not statistically significant, the GC rs7041 variant demonstrated a borderline trend (p = 0.084), suggesting a potential variation in genotype distribution across the diagnostic categories. For all other variants, genotype frequencies were similar, and broadly comparable genetic patterns were observed across the study population.

Given the relatively small sample size and the exploratory nature of the study, p-values were initially interpreted without adjustment for multiple testing. When the Bonferroni correction was applied (adjusted α = 0.05 / 8 = 0.00625), no association reached statistical significance (all adjusted p > 0.00625) (Table 3).

Overall, these findings indicate that vitamin D-related polymorphisms were largely similar among the allergy, Hashimoto’s thyroiditis, and ASD groups, with no distinct disease-specific genetic variation detected. Nonetheless, the borderline trend observed for GC rs7041 highlights a potential area of interest that warrants further investigation in larger populations with greater statistical power.

## Discussion

Serum 25-hydroxyvitamin D (25(OH)D) levels are influenced by environmental factors such as sunlight exposure, geographic latitude, ethnicity, age, sex, and dietary supplementation [10-12]. However, twin and family studies have demonstrated that 23%-80% of this variability is genetically determined [10,13]. Polymorphisms in genes involved in the synthesis, transport, and metabolism of vitamin D, particularly GC, CYP2R1, and VDR, have been shown to significantly affect serum 25(OH)D concentrations [14-16]. These genetic variants have also been associated with allergic diseases, autoimmune thyroid disorders, and autism. Therefore, identifying genetic variants that predispose individuals to vitamin D deficiency is crucial for early diagnosis and the development of personalized preventive strategies.

The CYP2R1 gene encodes the cytochrome P450 2R1 enzyme, which catalyzes the 25-hydroxylation of vitamin D [17]. The promoter variant rs2060793 has been linked to lower serum 25(OH)D levels, with the G allele associated with vitamin D deficiency [12,15,16]. Similarly, the TT genotype of rs1562902 has been correlated with reduced 25(OH)D levels [12,15]. In our study, these variants were more frequently observed among individuals with Hashimoto’s thyroiditis (Table 2).

The GC gene encodes the vitamin D-binding protein (DBP), which is responsible for the transport of vitamin D and its metabolites. The C allele of rs2282679 is associated with lower serum vitamin D concentrations [18-20], while the TT genotype of rs7041 has also been linked to vitamin D deficiency [19,21]. In our study, these variants were observed more frequently in the ASD group (Table 2).

The major biological effects of vitamin D are mediated through the VDR, a nuclear transcription factor. Four VDR polymorphisms, BsmI, ApaI, FokI, and TaqI, are among the most extensively studied [22]. A summary of the key vitamin D-related genetic variants reported in previous studies and their associations with vitamin D deficiency risk is presented in Table 4.

**Table 4:** Reported associations between CYP2R1, GC, and VDR gene variants and the risk of vitamin D deficiency. VDR: vitamin D receptor; CYP2R1: cytochrome P450 family 2 subfamily R member 1; GC: group-specific component (vitamin D–D-binding protein);SNP: single-nucleotide polymorphism​​​​​​*; *rs: reference SNP ID; A, T, C, G, nucleotide bases *For VDR polymorphisms, the classical genotype notations (FF, Ff, ff; BB, Bb, bb; TT, Tt, tt) correspond to modern genotype classifications, representing homozygous major, heterozygous, and homozygous minor genotypes, respectively.

Gene	rsID	Variant	Genotype	Risk Association	References
CYP2R1	rs2060793	A>G	G/C	GG/CC: High relative risk for low vitamin D levels. AG/CT: Moderate relative risk for low vitamin D levels. AA/TT: Reduced relative risk for low vitamin D levels.	[12,15,16]
CYP2R1	rs1562902	C>T	T/A	TT/AA: High relative risk for low vitamin D levels. CT/GA: Moderate relative risk for low vitamin D levels. CC/GG: Reduced relative risk for low vitamin D levels.	[12]
GC	rs2282679	T>G	G/C	GG/CC: High relative risk for low vitamin D levels. TG/AC: Moderate relative risk for low vitamin D levels. TT/AA: Reduced relative risk for low vitamin D levels.	[20]
GC	rs7041	A>C T>G	A/T	AA/TT: High relative risk for low vitamin D levels. AC/TG: Moderate relative risk for low vitamin D levels. CC/GG: Reduced relative risk for low vitamin D levels.	[19,21]
VDR (Taql)	rs731236	A>G (T>C) TT(TT) TC(Tt) CC(tt)	G/C	GG/CC: High relative risk for low vitamin D levels. AG/TC: Moderate relative risk for low vitamin D levels. AA/TT: Reduced relative risk for low vitamin D levels.	[23]
VDR (Bsml)	rs1544410	C>T (G>A) TT(BB) TC(Bb) CC(bb)	G/C	CC/GG: High relative risk for low vitamin D levels. CT/GA: Moderate relative risk for low vitamin D levels. AA/TT: Reduced relative risk for low vitamin D levels.	[24]
VDR (Fokl)	rs2228570	A>G (T>C) CC(FF) CT(Ff) TT(ff)	G/C	GG/CC: High relative risk for low vitamin D levels. AG/TC: Moderate relative risk for low vitamin D levels. AA/TT: Reduced relative risk for low vitamin D levels.	[25]
VDR (Apal)	rs7975232	C>A (G>T) TT(AA), TG(Aa) GG(aa)	C/G	CC/GG: High relative risk for low vitamin D levels. CA/GT: Moderate relative risk for low vitamin D levels. AA/TT: Reduced relative risk for low vitamin D levels.	[26]

The CC genotype of the VDR TaqI rs731236 variant has been reported to be associated with lower vitamin D levels (Table 4) [23]. In contrast, in our study, the heterozygous CT genotype was predominantly observed in the allergy and ASD groups (Table 2). The GG genotype of the BsmI rs1544410 polymorphism has been identified in the literature as being associated with vitamin D deficiency (Table 4) [24]; in our study, G allele carriage was notably high (Table 2). The GG genotype of the FokI rs2228570 variant is known to increase the risk of vitamin D insufficiency (Table 4) [25] and was the most common genotype across all groups in our analysis (Table 2). Furthermore, the CC genotype of the ApaI rs7975232 variant has been reported to be associated with the lowest serum vitamin D levels (Table 4) [24,26]. These variants showed differing frequencies among clinical subgroups in our study (Table 2).

Taken together, these findings suggest that genetic variations in vitamin D-related genes may differ among disease groups and potentially influence vitamin D metabolism.

Epigenetic mechanisms and the role of vitamin D

Our study demonstrated differing distributions of vitamin D-related genetic variants among disease groups. This finding aligns with evidence in the literature suggesting that vitamin D regulates gene expression not only through genetic polymorphisms but also via epigenetic mechanisms. Genes involved in vitamin D metabolism, VDR, CYP2R1, CYP27B1, and CYP24A1, contain methylation-sensitive CpG islands in their promoter regions, and transcriptional regulation is mediated through DNA methylation and histone modifications. Additionally, VDR interacts with chromatin-remodeling enzymes such as histone acetyltransferases (HATs) and histone deacetylases (HDACs) to modulate gene expression. Alterations in these epigenetic processes have been reported to affect immune responses, cellular development, and inflammatory regulation, and their dysregulation has been linked to several pathologies, including cancer, osteoporosis, and cardiovascular diseases [27].

Environmental and dietary factors can also influence these epigenetic pathways. In an experimental model using a high-fat diet, hypermethylation of CYP2R1, VDR, CYP27A1, and CYP27B1 genes, and hypomethylation of CYP24A1 were observed. These findings suggest that unbalanced nutrition may adversely affect vitamin D metabolism through epigenetic modifications [28].

Effects of VDR polymorphisms on VDR methylation

A study investigating VDR methylation during pregnancy reported that the duration of light exposure (photoperiod) accounted for approximately 7% of methylation variability. The same study also demonstrated that VDR polymorphisms influence methylation patterns; specifically, the C alleles of TaqI and FokI polymorphisms were associated with decreased methylation levels. These findings reflect the complex interplay between environmental factors, vitamin D metabolism, and genetic regulation [29].

In our study, the findings related to VDR FokI and TaqI variants observed in Hashimoto’s thyroiditis (FokI: 90%, TaqI: 50%), allergic diseases (FokI: 92.1%, TaqI: 71.1%), and ASD (FokI: 91.7%, TaqI: 66.7%) suggest that environmental and immunological factors play important roles in these conditions. This observation supports the notion that such clinical entities may differ at both genetic and epigenetic levels.

Methylation effects of the VDR TaqI polymorphism

The VDR TaqI polymorphism (rs731236) is located within a CpG island, where the genotype can determine the presence or loss of a CpG site. This variant can influence both local methylation and methylation of the CGI 1060 region at the 3′ end of the VDR gene. When the CpG site is present, methylation occurs and may subsequently reduce local methylation within the CGI 1060 region. These methylation alterations have been reported to affect 3′ promoter activity, long non-coding RNA (lncRNA) transcription, and the post-transcriptional regulation of VDR expression. The C allele is associated with hypermethylation, which may lead to lower lncRNA levels, reduced VDR repression, and increased gene expression. Conversely, the T allele is linked to hypomethylation, elevated lncRNA levels, and suppressed VDR expression [30].

In our study, the TaqI (T) allele carriage was found to be high across all disease groups: 92.1% in the allergy group (TT + CT), 80.0% in the Hashimoto’s thyroiditis group (TT + CT), and 83.3% in the ASD group (TT + CT) (Table 2). These findings suggest that T allele carriage may indicate altered VDR activity within these clinical populations.

Effects of VDR polymorphisms on Hashimoto’s thyroiditis

The VDR is expressed in various immune cells, including T and B lymphocytes, dendritic cells, macrophages, and neutrophils. Through VDR-mediated mechanisms, vitamin D suppresses the production of proinflammatory cytokines such as IL-1, IL-6, IL-12, and TNF-α, while enhancing the expression of the anti-inflammatory cytokine IL-10. It also promotes the proliferation of immunosuppressive regulatory T cells (Tregs) and inhibits B-cell differentiation and antibody production [31].

In Hashimoto’s thyroiditis, dysregulation of these mechanisms may enhance autoimmune processes. Meta-analytic data have shown that the FokI G allele and GG genotype increase the risk of Hashimoto’s thyroiditis, whereas TaqI, ApaI, and BsmI polymorphisms were not significantly associated [32]. In our study, the FokI GG genotype was found in 60% and the AG genotype in 30% of individuals with Hashimoto’s thyroiditis (combined AG+GG: 90%), making this the most prevalent variant. High carriage rates were also observed for other polymorphisms (ApaI: 50%, BsmI: 80%, TaqI: 50%) (Table 2).

Effects of VDR polymorphisms on autism

Variants in the VDR gene have been associated with neurodevelopmental disorders. Systematic reviews have reported that the C allele of TaqI rs731236 may increase the risk of ASD [33]. In our study, this variant was also found at a high frequency in the ASD group (CT+CC: 66.7%). Elevated carriage rates were also observed for other polymorphisms linked to vitamin D deficiency: ApaI (75%), BsmI (83.3%), and FokI (91.7%) (Table 2). These findings suggest that the FokI, ApaI, TaqI, and BsmI variants may represent potential genetic risk factors for autism.

Effects of VDR polymorphisms on allergic diseases

For VDR polymorphisms, the risk allele associated with vitamin D deficiency is the C allele for ApaI and TaqI (Table 4) [23,26]. However, a meta-analysis reported the A allele of ApaI and the T allele of TaqI as risk alleles for allergy. The same analysis indicated that ApaI (A), BsmI (G), and TaqI (T) variants were associated with allergy susceptibility in certain populations, while no significant association was found for FokI (G) [34]. In contrast, our study revealed high carriage frequencies for the risk alleles of all four variants in the allergy group: ApaI (89.5%), BsmI (86.8%), TaqI (92.1%), and FokI (92.1%) (Table 2).

These results suggest that variations in the VDR gene may play a role in modulating immune function and influencing the development of allergic responses.

Effects of VDR polymorphisms on the microbiota

Vitamin D plays a crucial role in maintaining gut health and controlling inflammation by regulating host-microbiota interactions [35]. Through VDR-mediated mechanisms, it modulates both innate and adaptive immune responses, supports epithelial barrier integrity, and enhances the production of antimicrobial peptides [35,36]. The VDR TaqI TT genotype has been associated with increased microbial diversity and a higher abundance of short-chain fatty acid-producing bacteria [37]. In our study, the TT genotype was observed at varying frequencies across the allergy, ASD, and Hashimoto’s thyroiditis groups (Table 2).

The gut microbiota can also influence VDR expression and activity. Supplementation with vitamin D in combination with probiotics such as *Lactobacillus rhamnosus* GG, *Lactobacillus plantarum*, and *Bifidobacterium *BB-12 has been shown to increase intestinal VDR expression and serum vitamin D levels. Moreover, vitamin D-related genetic polymorphisms may shape microbiota composition, while gut bacteria can, in turn, modulate VDR expression and serum vitamin D levels [38].

These findings suggest that VDR genetic variants, together with inadequate vitamin D intake, may jointly affect microbiota balance and immune function. Thus, the reciprocal interactions among genetic variants, environmental factors, and the microbiota may help explain the biological differences observed in these clinical conditions.

Strengths and limitations

One of the major strengths of this study is that it is among the few to evaluate vitamin D within an integrative framework that simultaneously considers genetic, epigenetic, and microbiota-related factors. This multidimensional approach provides novel perspectives for preventive medicine and offers valuable public health implications.

However, the main limitation of this study is the relatively small sample size, which may restrict the generalizability of the findings. Since the majority of participants were women (73%), a potential bias may have been introduced in genotype distribution results, particularly regarding thyroid disorders that are more prevalent among females. This factor should be taken into account when interpreting the results. Larger, multicenter studies with greater statistical power are needed to confirm and expand upon these findings.

## Conclusions

This study proposes the hypothesis that polymorphisms in genes related to vitamin D metabolism may have adverse effects in terms of epigenetic regulation and microbial dysbiosis. The findings indicate that these genetic variants may influence epigenetic mechanisms and microbiota diversity, thereby contributing to the pathophysiology of autoimmune, neurodevelopmental, and allergic diseases. Considering that genetic and epigenetic processes involved in vitamin D metabolism are affected by nutritional factors, nutrigenetic-based approaches and microbiota modulation hold significant potential for supporting vitamin D function, maintaining overall health, and preventing disease.

While previous studies have generally evaluated vitamin D levels only through serum concentrations, this research simultaneously examines genetic, epigenetic, and microbiota interactions, offering an integrative perspective. This approach underscores the importance of a multidimensional evaluation of vitamin D deficiency for developing effective preventive and therapeutic strategies. In the future, investigating the epigenetic regulation patterns and microbiota interactions of vitamin D-related genetic variants in larger populations will provide a scientific foundation for the development of personalized nutrition and treatment protocols. Moreover, this study may provide clinicians with a broader perspective on the multifactorial nature of vitamin D deficiency, helping to promote more preventive and individualized approaches in clinical practice.
